# A novel rabbit derived anti-HER2 antibody with pronounced therapeutic effectiveness on HER2-positive breast cancer cells in vitro and in humanized tumor mice (HTM)

**DOI:** 10.1186/s12967-020-02484-9

**Published:** 2020-08-15

**Authors:** Anja Kathrin Wege, Nicole Kirchhammer, Linda Veronique Kazandjian, Sandra Prassl, Michael Brandt, Gerhard Piendl, Olaf Ortmann, Stephan Fischer, Gero Brockhoff

**Affiliations:** 1 Clinic of Gynecology and Obstetrics, University Medical Center Regensburg, Regensburg, Germany; 2 Department of Biomedicine, University of Basel, Basel, Switzerland; 3 MAB Discovery GmbH, Polling, Germany

**Keywords:** Anti-HER2 antibody, Breast cancer, Treatment efficiency, Humanized tumor mice, Monoclonal antibody

## Abstract

**Background:**

Antibody based cancer therapies have achieved convincing success rates combining enhanced tumor specificity and reduced side effects in patients. Trastuzumab that targets the human epidermal growth factor related receptor 2 (HER2) is one of the greatest success stories in this field. For decades, trastuzumab based treatment regimens are significantly improving the prognosis of HER2-positive breast cancer patients both in the metastatic and the (neo-) adjuvant setting. Nevertheless, ≥ 50% of trastuzumab treated patients experience *de*-*novo* or acquired resistance. Therefore, an enhanced anti-HER2 targeting with improved treatment efficiency is still aspired.

**Methods:**

Here, we determined cellular and molecular mechanisms involved in the treatment of HER2-positive BC cells with a new rabbit derived HER2 specific chimeric monoclonal antibody called “B100″. We evaluated the B100 treatment efficiency of HER2-positive BC cells with different sensitivity to trastuzumab both in vitro and in the presence of a human immune system in humanized tumor mice.

**Results:**

B100 not only efficiently blocks cell proliferation but more importantly induces apoptotic tumor cell death. Detailed in vitro analyses of B100 in comparison to trastuzumab (and pertuzumab) revealed equivalent HER2 internalization and recycling capacity, similar Fc receptor signaling, but different HER2 epitope recognition with high binding and treatment efficiency. In trastuzumab resistant SK-BR-3 based humanized tumor mice the B100 treatment eliminated the primary tumor but even more importantly eradicated metastasized tumor cells in lung, liver, brain, and bone marrow.

**Conclusion:**

Overall, B100 demonstrated an enhanced anti-tumor activity both in vitro and in an enhanced preclinical HTM in vivo model compared to trastuzumab or pertuzumab. Thus, the use of B100 is a promising option to complement and to enhance established treatment regimens for HER2-positive (breast) cancer and to overcome trastuzumab resistance. Extended preclinical analyses using appropriate models and clinical investigations are warranted.

## Background

The receptor tyrosine kinase Human Epidermal Growth Factor Receptor 2 (HER2) is a driver of breast carcinogenesis and progression. It plays a pivotal role in the regulation of cell proliferation and its downstream signaling pathways are involved in the regulation of migration, differentiation, and apoptosis [1]. HER2 is overexpressed in about 20 % of all breast cancers (BC), mostly caused by HER2 gene amplification,  and has been associated with a worse prognosis and a higher risk of relapse [2]. In contrast to its molecular relatives HER1, HER3, and HER4 and due to its unique extracellular 3D conformation, the HER2 receptor does not bind any known native ligand. Receptor activation occurs mainly by homodimerization- and heterointeraction with other receptor family members. Upon activation, the Ras-MAPK pathway triggers tumor cell proliferation and the mTOR signaling promotes cell survival by counteracting pro-apoptotic signals [3]. Trastuzumab (Herceptin™), initially generated by Genentech Inc. (San Francisco, CA, USA) as monoclonal antibody “4D5”, is a recombinant humanized monoclonal IgG1 antibody that binds to the extracellular domain IV of the HER2 protein close to the cell membrane [4]. Upon binding to HER2 [5] trastuzumab ligand independently affects homo-dimerization, triggers antibody dependent cellular cytotoxicity (ADCC) by activating CD16-positive immune cells, and it prevents shedding of the extracellular receptor domain [3] which otherwise would cause a constitutively hyper-activated p95-HER2 domain [6]. However, ≥ 50% of patients with HER2-positive breast cancer don’t show response either ab initio or acquire resistance during treatment [7]. Accordingly, there is still a significant high rate of cancer related death amongst the group of HER2-positive BC patients [8]. Another HER2-specific humanized monoclonal antibody pertuzumab (Perjeta™, Genentech Inc.) recognizes a rather distal located extracellular HER2 epitope and became clinically approved in combination with trastuzumab for the treatment of BC patients in the neoadjuvant and adjuvant setting. Pertuzumab complements the trastuzumab mediated therapeutic effects predominantly by preventing HER2 (hetero-)dimerization and receptor phosphorylation [9–11]. The application of both trastuzumab and pertuzumab in combination (but not the use of pertuzumab alone) enhanced the pathological complete response rates (pCR) from 29 to 46% with locally advanced, inflammatory, or early-stage HER2-positive breast cancer as demonstrated by the NeoSphere2 trial [12] . Nevertheless, long term monitoring is still needed to assess the capability of the two antibody based treatment regimens to delay or even prevent distant relapse and to determine the overall survival benefit. Apparently, the combined use of clinically active therapeutic anti-HER2 antibodies is a very powerful strategy to further improve the course (and outcome) of HER2-positive BC disease, whereby an additive or even synergistic treatment effect has been attributed to different but complementing molecular mechanisms triggered by two immunoglobulins [13, 14].

So far, most of the monoclonal antibodies approved for clinical application have been generated in mice. However, mouse strains typically used for immunization and antibody generation are usually inbred, are housed under specific pathogen-free conditions and the number of B cells, which can be isolated from the small spleen, is limited. In contrast, rabbits have a larger spleen and a competent immune system inclusive B cells which produce antibodies with enhanced affinity and a wide spectrum of specificity [15, 16]. More specifically, the rabbit’s immune system recognizes a greater variety of epitopes including small proteins, which are not immunogenic in mice. This stronger immune response is due to a special mechanism that underlies the generation of a large antibody repertoire. Identical to human and mice rabbits build their antibodies by a rearrangement of so called variable, diversity, and joining, (VDJ) gene segments as well as somatic hypermutation. However, in rabbits (and chicken) somatic gene conversion additionally occurs in the appendix and other Gut-Associated Lymphoid Tissues (GALT), which ensures not only a wider antibody repertoire but also results in a higher antibody binding affinity [16, 17]. Important steps in the generation and utilization of monoclonal antibodies were the discovery of the rabbit plasmocytoma cell line 240E for fusions [18] that enabled the generation of FDA-approved in vitro diagnostic tools (e.g., anti-HER2, anti-PD-L1). Moreover, 240E derived therapeutic humanized rabbit monoclonal antibodies (mAbs) as for example sevacizumab (anti VEGF) and APX005M (anti CD40) are currently being tested in clinical trials. MAB Discovery GmbH (MAB) has employed a fully automated high throughput platform for rabbit immunization, isolation of specific B cells from blood instead of spleen, expansion of the B cell clones, and sequencing of the monoclonal antibodies. With the help of this technique MAB produced a variety of rabbit based humanized anti-HER2 antibodies, which we characterized in vitro. A number of newly generated anti-HER2 antibodies, amongst them a clone that we named “B100”, shows in vitro inhibition of tumor cell proliferation and upon binding to target cells even induces tumor cell apoptosis to a significant extent. Selected anti-HER2 rabbit derived mAbs were tested in vivo using humanized tumor mice (HTM), a mouse model in which human immune system and human tumor growth coexist [19]. In particular, we utilized a well characterized HER2-positive SK-BR-3 based HTM model, which was proven to represent as trastuzumab resistant mouse model [20]. This human-like mouse model has been previously used for treatment studies [20, 21] and to improve diagnostic procedures [22].

Overall, we generated and characterized a rabbit derived, chimeric, highly efficient anti-HER2 mAb called B100 that comes with a pronounced pro-apoptotic capacity on HER2 positive BC cells. The treatment efficiency in HTM was superior compared to trastuzumab and pertuzumab treatments due to reduced primary tumor growth, tumor cell dissemination and metastasis.

## Materials and methods

### Immunization, selection and chimerization of HER2-specific antibodies

The generation of chimerized rabbit derived monoclonal antibodies was previously described [23]. In brief, human recombinant protein was used as immunogen for wild-type albino zika rabbit immunization. Rabbit primary antibodies were derived from B cell clones from peripheral blood. Extracted antibody coding DNA of the variable regions were sequenced. The isolated and codon modified (i.e., chimerization by a human derived Fc sequence) IgG1 monoclonal anti-HER2 antibody was produced in HEK293-FreeStyle cells (ThermoFisher Scientific, Waltham, MA, USA). Antibody purification out of cell supernatants was accomplished in two steps using the “ÄKTA Avant” purification system (GE Healthcare, Munich, Germany). The antibodies were purified by affinity chromatography using a Protein A resin (MabSelect SuRe, GE Healthcare), followed by a preparative size exclusion chromatography (MAB Discovery GmbH).

### ELISA binding assay

384-well Maxisorp microtiter plates (ThermoFisher Scientific) were coated with 12.5 μl of coating protein that was 0.5 μg/ml solution diluted to the desired concentration of recombinant human ErbB2/HER2 Fc (R&D Systems, Minneapolis, MN, USA). After incubation for 60 min at room temperature plates were washed three times with PBS washing buffer containing 0.05% Tween-20 (Sigma-Aldrich Merck KGaA, Darmstadt, Germany). 90 μl blocking buffer PBS supplemented with 0.05% Tween-20 plus 2% BSA (Roche Molecular Systems, Mannheim, Germany) was added and incubated for 60 min at room temperature followed by three times washing with 90 μl washing buffer. Then, 12.5 μl of the primary antibody solution (PBS, 2% BSA and 0.05% Tween 20) was added and incubated for 60 min at room temperature followed by three washing steps. Next, 12.5 μl of detection antibody anti-human F_ab2_ POD Antibody STAR126P (AbD Serotec Biorad, Puchheim, Germany) diluted 1:1000 in PBS, 2% BSA and 0.05% Tween 20 was added and incubated for 60 min at room temperature. After three times washing 15 μl of TMB solution (ThermoFisher Scientific) was added and developed until a stable signal was obtained. The incubation was stopped by addition of 15 μl of 1 M Titripur Hydrochloric Acid solution (Merck KGaA, Darmstadt, Germany). The absorbance was determined at 450 nm/620 nm and data were analyzed with Excel Fit (Fit Model 205, pre-fit for all 4 parameters, no constraints on any parameter, EC50 = parameter C).

### Fcγ receptor signaling

Fcγ receptor signaling was analyzed using a BioGlo™ Luciferase ADCC Reporter Bioassay (Promega GmbH, Walldorf, Germany, cat. # G7102). The commercially available assay contains engineered Jurkat cells (derived from the clone E6-1; ATCC # TIB-152) stably expressing the high affinity (V158) FcγRIIIa receptor variant and a response element called “nuclear factor of activated T-cells” (NFAT) that drives the expression of firefly luciferase as effector cells (name of GMO: Jurkat_pGL4.14-luc2-NFAT-Re-Hygro_pF9A-FcγRIIIa). Upon binding to target cells antibody biological activity on effector cells (i.e., ADCC) is quantified through the luciferase produced as a result of NFAT pathway activation. Luciferase activity in effector cells is quantified by measuring luminescence. Technically, target cells (SK-BR-3, 2500 cells/well; JIMT-1, 7500 cells/well; ZR-75-1, 15,000 cells/well) were cultured overnight on a 384-well assay plate (Corning Inc.). Luciferase assay reagent and ADCC assay buffer were prepared according to the manufactures instruction. Primary antibodies (B100, trastuzumab) were diluted in ADCC assay buffer (conc.: 2.5–5000 ng/ml) and added to each well. ADCC buffer without antibody was used as zero control. Frozen stored effector cells (Jurkat, 4000 cells/well) were thawed in a water bath at 37 °C and added to each well. After an incubation of 6 h at 37 °C, 5% CO_2_, Bio-Glo Luciferase Assay Reagent was added and luminescence was measured according to the BioGlo™ assay manufacturer’s instructions.

### Epitope competition assay

Goat anti-human IgG (500 ng/ml, Sigma-Aldrich) was coated for 60 min at room temperature using 96 well plates. After three times of washing, plates were blocked for 60 min, washed again (3×) and the primary antibodies (pertuzumab, trastuzumab; 1 mg/ml) were added and incubated for 60 min. The competing antibody (trastuzumab, pertuzumab, A57, B100, or B106; 2.5–5000 ng/ml), the target protein HER2-HIS (R&D Systems; conc. 0.1 mg/ml), and the detection antibody anti-HIS-POD (Sigma Aldrich Merck, conc. 7.5 mg/ml) were mixed together and incubated for 60 min at room temperature. The pre-incubated mixture was subsequently added to the plate and incubated for another 60 min, followed by three time washing, TMB (ThermoFisher Scientific) incubation and reading was done at 450/620 nm.

### HER2 Internalization assay

To exclusively analyze the internalization of the HER2 receptor after treatment with trastuzumab, pertuzumab, B100, B106 or A57, receptor recycling was inhibited with Monensin. Cells were pre-incubated with 2 µM Monensin for 2 h in DMEM media (5% FCS). Afterwards, cells were incubated with 10 µg/ml of the treating antibody for 0, 0.5, 1, 3, 5, and 21 h in DMEM media (5% FCS) at 37 °C to allow antibody mediated receptor internalization. Finally, 0.25 × 10^6^ cells were incubated with a non-competitive fluorescein iso-thiocyanate (FITC) conjugated mouse anti human HER2 antibody (10 µg/ml, clone 24D2, BioLegend, San Diego, CA, USA) on ice for 30 min. Cells were analyzed by flow cytometry at different time points. A decrease of cell surface HER2 by increasing treatment duration indicated antibody dependent internalization.

### HER2 antibody complex recycling

The recycling assay is based on fluorescence quenching of Alexa Fluor 488 by an anti-Alexa Fluor 488 antibody [24]. 100 µg trastuzumab, pertuzumab and B100 were conjugated with Alexa Fluor488 using an Alexa Fluor 488 antibody labeling kit (ThermoFisher). Saturated concentrations of labeled antibodies were determined using a titration curve. Cells were pulsed with 10 µg/ml conjugated trastuzumab, pertuzumab or B100 in pre-warmed DMEM media with 5% FCS at 37 °C for 30 min to allow receptor-mediated internalization of the antibody-receptor complex. To quench the cell surface Alexa 488 fluorescence signal, the cells were pulsed with 50 µg/ml of a polyclonal anti-Alexa Fluor 488 antibody (ThermoFisher) for 25 min on ice. After washing, the cells were incubated with pre-warmed media containing 20 µg/ml anti-Alexa Fluor 488 at 37 °C for a period of 0, 7.5, 15, 22.5 and 30 min. During a possible recycling of the antibody-receptor complex back to the plasma membrane the signal of the recycled antibody (conjugated with Alexa Fluor 488) was quenched by the anti-Alexa Fluor 488 antibody solved in culture medium. At each time point the fraction of antibody retained in cells was calculated by normalizing the fluorescence intensity of Alexa Fluor 488 with the signal to the 0 min time point. As a negative control, cells were pre-incubated with the recycling inhibitor Monensin (2 µM) for 1 h which was added at all incubation steps. The recycling quenching step was performed on ice to prevent recycling.

### Breast cancer cell lines and treatments

All BC cell lines used in this study were obtained from the American Type Culture Collection (ATCC, LGC Standards, Wesel, Germany). Cells were incubated in Dulbecco’s modified Eagle’s medium (DMEM) supplemented with 5% fetal calf serum (FCS) (both PAA Laboratories, Pasching, Austria) under standard cell culture conditions. Later on after the purchase cell lines were authenticated by a nanoplex PCR based STR profiling (DSMZ, Braunschweig, Germany). SK-BR-3 cells were used to generate HTM (see below). Cells were treated in vitro with trastuzumab and pertuzumab (both made available by MAB) and chimeric mAbs generated by MAB using 2 µg/ml, respectively. In combination treatments with two mAbs only 1 µg/ml of each mAb was applied which ensures an equivalent total antibody concentration of 2 µg/ml. Untreated cells served as control samples.

### Flow cytometry analysis

All flow cytometric measurements were performed on a FACSCanto II flow cytometer (BD Biosciences, San Jose, CA) equipped with a blue (488 nm), violet (405 nm), and red (633 nm) laser and standard optical configuration. Data were analyzed with FACSDiva Software v7.0 (BD Biosciences).

- Proliferation assessment: Upon harvesting by trypsinization cells were washed twice with PBS, fixed and permeabilized by an incubated in cooled MeOH (70%) overnight. Afterwards the cells were washed twice with PBS (supplemented with 1% FBS and 0.01% NaN_3_) to remove the alcohol, incubated for 20 min in the presence of RNAase (Roche Molecular Systems) at 37 °C and finally stained with 1 μg/ml DAPI prior to analysis. Cell doublets, aggregates, and debris were excluded by pulse processing and DNA histograms of the gated population were plotted on a linear scale. Cell cycle fractions (% of cells in G0/G1-, S- and G2/M-phase) were quantified using the ModFit LT 3.2 software (Verity Software House, Topsham, ME, USA).
- Annexin V‐FITC/DAPI Assay: Cells were grown for 72 h in 5% FCS/DMEM and treated for 72 h with trastuzumab, pertuzumab, or mAb (MAB). Cells were harvested by trypsinization without discarding the supernatant. As a positive apoptotic control, cells were treated with 10 μM Camptothecin for 17 h. Untreated cells served as negative control. Cells were stained immediately after harvesting using the TACS Annexin-V‐FITC Apoptosis Detection Kit (ImmunoTools, Friesoythe, Germany) according to the manufacturer’s instructions. Single‐color controls (unlabeled, Annexin-V‐FITC only, DAPI only cells) were used in combination with double‐stained samples (Annexin-V‐FITC/DAPI-stained cells) to set up compensation controls.
- Analyzing the reconstitution of immune cells of humanized mice: Organs (spleen, lung) were dissociated by passing the cells through 40 μm cell strainer (BD Biosciences). Bone marrow (BM) cells were collected from the femur by clipping the ends and flushing the bone cavity with 10 ml PBS using a syringe with a 27 G needle (BD Biosciences).To reduce non-specific binding, cells were incubated with 1% mouse serum for 10 min. Samples were stained using the following mAb: anti-CD3-FITC (clone HIT3a), anti-CD19-PE (clone HIB19), anti-CD45-APC (clone HI30), anti-NKp46-PE (clone 9-E2), anti-CD4-APC-H7 (clone SK3), anti-CD8-PE (clone HIT8a), and anti-CD56-Horizon™V450 (clone B159) (all BD Biosciences). In addition, we used anti-CD33-PerCP-Cy5.5 (clone WM53) and anti-CD16-PE (clone 3G8) (BioLegend). In advance all antibodies were titrated to an optimal concentration. Appropriate mouse immunoglobulin antibodies were used as isotype controls for all staining.
- Tumor cell phenotyping: Non-specific binding, was blocked by incubating the cells in 1% mouse serum for 10 min. Samples were stained using the following antibodies: anti-HER2-PE (clone NEU 24.7, BD Biosciences), anti-EPCAM BV421 (clone 9C4), anti-CD24-AF647 (clone ML5), and anti-CD44 AF488 (clone IM7) (the latter from BioLegend). Appropriate mouse immunoglobulin antibodies were used as isotype controls for all staining.

### Mice

NOD.*Cg*-*Prkdc*^*scid*^*Il2rγ*^*tm1Wjl*^*/SzJ* (NSG) mice were obtained from Jackson Laboratories and bred and kept in a specialized pathogen-free facility at the University of Regensburg. Humanized tumor mice were generated as previously described [19, 20]. Briefly, neonatal mice were irradiated (1 Gy) and 3 h later transplanted with 2–2.5 × 10^5^ human CD34^+^ cells isolated from umbilical cord blood (CB) using immunomagnetic beads (Miltenyi Biotech, Bergisch Gladbach, Germany) together with 3 × 10^6^ SK-BR-3 tumor cells. Important to mention is that mice transplanted with the same CB sample were split into different treatment and control groups. In all experiments, cells were co-transplanted into the liver of newborn mice. In the age of 9 weeks SK-BR-3 transplanted littermates (transplanted with the same CB) of HTM and TM littermates were divided into the different groups and treated with MAB antibodies (5 mg/kg/week i. p.) for 12 weeks. Animals were sacrificed and analyzed either at an early time point i.e., 9 weeks post-transplant, or at the age of 3 to 5 months.

The local veterinary authorities of the district government of Bavaria (Germany) approved all animal work (permission no. 54-2532.1-44/13). Cord blood samples were taken based on the approval given by the Ethics Committee of the University of Regensburg (permission no. 15-101-0057). All patients included in the study provided written informed consent.

### Immunohistochemistry

Tissue specimens (tumor, spleen, liver, brain, and lung) were prepared as previously described [19, 20]. Briefly, samples were fixed with 4% formalin and embedded in paraffin. Four µm slides were prepared, deparaffinized and stained with anti-HER2 rabbit polyclonal A0485 (Dako GmbH, Jena, Germany) automatically on a Ventana Nexes autostainer (Ventana, Tucson, USA) by using the streptavidin–biotin–peroxidase complex method and 3,3’-diaminobenzidine. All lung, liver, and brain specimens were analyzed for the number and distribution of HER2-positive tumor cells and scored as outlined in Table 1. The autostainer was programmed based on the instructions provided with the iView DAB detection kit (Ventana). Histological specimens were imaged with an AxioImager Z1 microscope (Zeiss, Oberkochen, Germany).

**Table 1:** Immunohistological scoring of lung metastases in HTM and TM

Score	Description
0	No tumor cells
1	1–5 tumor cells
2	Single cells distributed throughout organ
3	Cell cluster/metastases formation

SK-BR-3 transplanted HTM and TM were immunohistologically stained using anti-HER2 antibodies and tumor cells scored as indicated (count of tumor cells/high power field)

### Statistical analyses

All results are shown as mean ± SEM. All reported p-values were two-sided. p-values less than 0.05 were considered significant. For group wise comparison a one-way or two-way analysis of variance (ANOVA) with Dunnett’s post hoc test or Tukey’s multiple comparisons were applied and the tests are indicated in each figure and table legend. All statistical analyses were performed using GraphPad Prism (Ver. 6, GraphPad Software, La Jolla, CA, USA).

## Results

### Rabbit derived chimeric monoclonal anti-HER2 mAbs show pronounced anti-proliferative and pro-apoptotic activity

A total of 70 different anti-HER2 mAbs were tested in vitro for their efficacy in inhibiting cell proliferation and inducing apoptosis of SK-BR-3 (strong HER2 gene amplification, HER2/CEP17 ratio: 4.8, HER2 protein overexpression, moderately trastuzumab sensitive), MDA-MB-361 (moderate HER2 gene amplification, HER2/CEP17 ratio: 3.6, intermediate HER2 expression, trastuzumab insensitive), and MDA-MB-453 (moderate HER2 gene amplification, borderline HER2 gene ratio 2.1, intermediate HER2 protein expression, trastuzumab insensitive) BC cell lines.

**Fig. 1:**
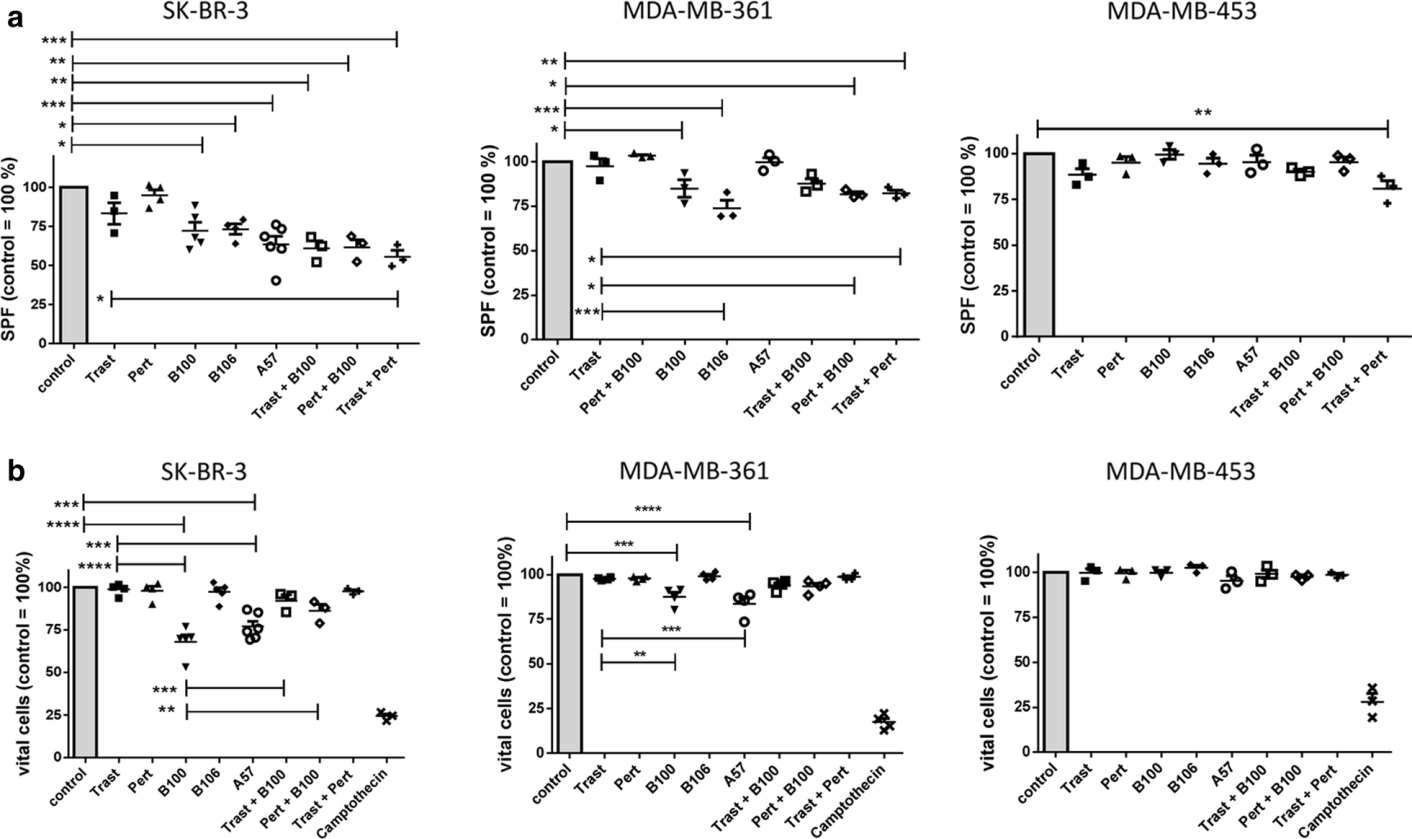
Treatment efficiency of different anti-HER2 antibodies in vitro. **a** SK-BR-3, MDA-MB-361, and MDA-MB-453 were treated with a total concentration of 2 µg/ml for 72 h and S-phase fraction (SPF, **a**) or apoptosis **b** in comparison to untreated control cells is displayed. Untreated controls were set 100% and differences from the control are shown for each antibody treatment. Significant differences are only indicated in relation to control and trastuzumab (**a**, **b**) or B100 (**b**) treatment. Tukey’s multiple comparisons test was applied and significances are indicated (*p < 0.05; **p < 0.01; ***p < 0.001; ****p < 0.0001). Trast, trastuzumab; Pert, pertuzumab

The S-phase fractions (SPF) of treated compared to untreated control cells (100%) were quantitatively determined by flow cytometry (Fig. 1a). Of note, the average SPFs of untreated cells were 15.34% (mean ± 1.23 SEM; SK-BR-3), 21.85% (mean ± 5.01 SEM; MDA-MB-361), and 27.39% (mean ± 0.74 SEM; MDA-MB-453). A treatment neither with trastuzumab nor with pertuzumab significantly reduced the SPF in in all three cell lines (Fig. 1a). However, when trastuzumab and pertuzumab were applied in combination a strong reduction of the SPF was observed in SK-BR-3 compared to untreated (p < 0.001) and to trastuzumab only treated cells (p < 0.05) as well as in MDA-MB-453 treated compared to untreated (p < 0.01) cells. Interestingly, the mAb A57 showed the strongest effect in SK-BR-3 compared to control (p < 0.001) followed by B106 (p < 0.05), and B100 (p < 0.05). On MDA-MB-361 cells, B100 (p < 0.05), B106 (p < 0.001) and the combined treatment with pertuzumab plus B100 (p < 0.05) as well as trastuzumab plus pertuzumab (p < 0.01) significantly reduced the SPF. Neither the addition of trastuzumab nor of pertuzumab to the B100 treatment did significantly enhance the treatment efficacy of B100. The weakest treatment efficiency was seen with MDA-MB-453. These cells did just slightly respond to the trastuzumab/pertuzumab treatment (Fig. 1a).

In addition we determined the fraction of apoptotic cells by flow cytometry upon mAb treatments compared to untreated cells (Fig. 1b). Vital cells of untreated cells were normalized to 100%, respectively. The average percentage of live cells in the untreated controls were 88.9% (mean ± 0.42 SEM; SK-BR-3), 91.9% (mean ± 0.5 SEM; MDA-MB-361), and 89.53% (mean ± 0.48 SEM; MDA-MB-453). In SK-BR-3, the treatment with B100 (p < 0.0001) and A57 (p < 0.001) significantly decreased the fraction of vital cells compared to untreated cells. The strong pro-apoptotic effect of the B100 and A57 treatment became particularly apparent in comparison to the trastuzumab and pertuzumab treated SK-BR-3 cells (Fig. 1b left). Trastuzumab and pertuzumab treatments did not result in any amount of apoptotic cells. Interestingly, the B100 treatment alone significantly reduced the fraction of vital cells more efficiently than in combination with trastuzumab (p < 0.001) or pertuzumab (p < 0.01). This phenomenon was mainly obvious in SK-BR-3 cells. The reduced amount of apoptotic cells in B100/Trast and B100/Pert treated cells compared to cells treated with B100 only is probably due to the half concentrated mAbs (i.e., 1 µg/ml, respectively) used in combination treatments. Western blotting revealed an induction of caspase 3 and cytochrome c protein whereas survivin was decreased in B100 treated SK-BR-3 compared to trastuzumab, and pertuzumab treated cells (data not shown). In MDA-MB-361 cells B100 as well as A57 treatments significantly reduced the fraction of vital cells compared to untreated and to trastuzumab treated cells (Fig. 1b middle). There was no additive effect when B100 was applied simultaneously with trastuzumab or with pertuzumab. In addition, there was no effect of any antibody incubation detectable in MDA-MB-453 cells (Fig. 1b right).

### B100 shows no epitope competition to trastuzumab but similar internalization, recycling and FcR signaling capacity

In a dose response ELISA assay B100 binding revealed an EC50 of 5.2 ng/ml which is in the same range as the EC50 values of trastuzumab (3.0 ng/ml) and pertuzumab (4.7 ng/ml). Fitting curves are shown in Figure S1 and reflect sigmoid antibody binding characteristics for all three monoclonals. The goodness of fit were for B100 0.8412, for Trast 0.7954, and for Pert 0.8286. EC50 values for binding activities on SK-BR-3 cells were 78 ng/ml (B100), 153 ng/ml (trastuzumab), and 130 ng/ml (pertuzumab).

**Fig. 2:**
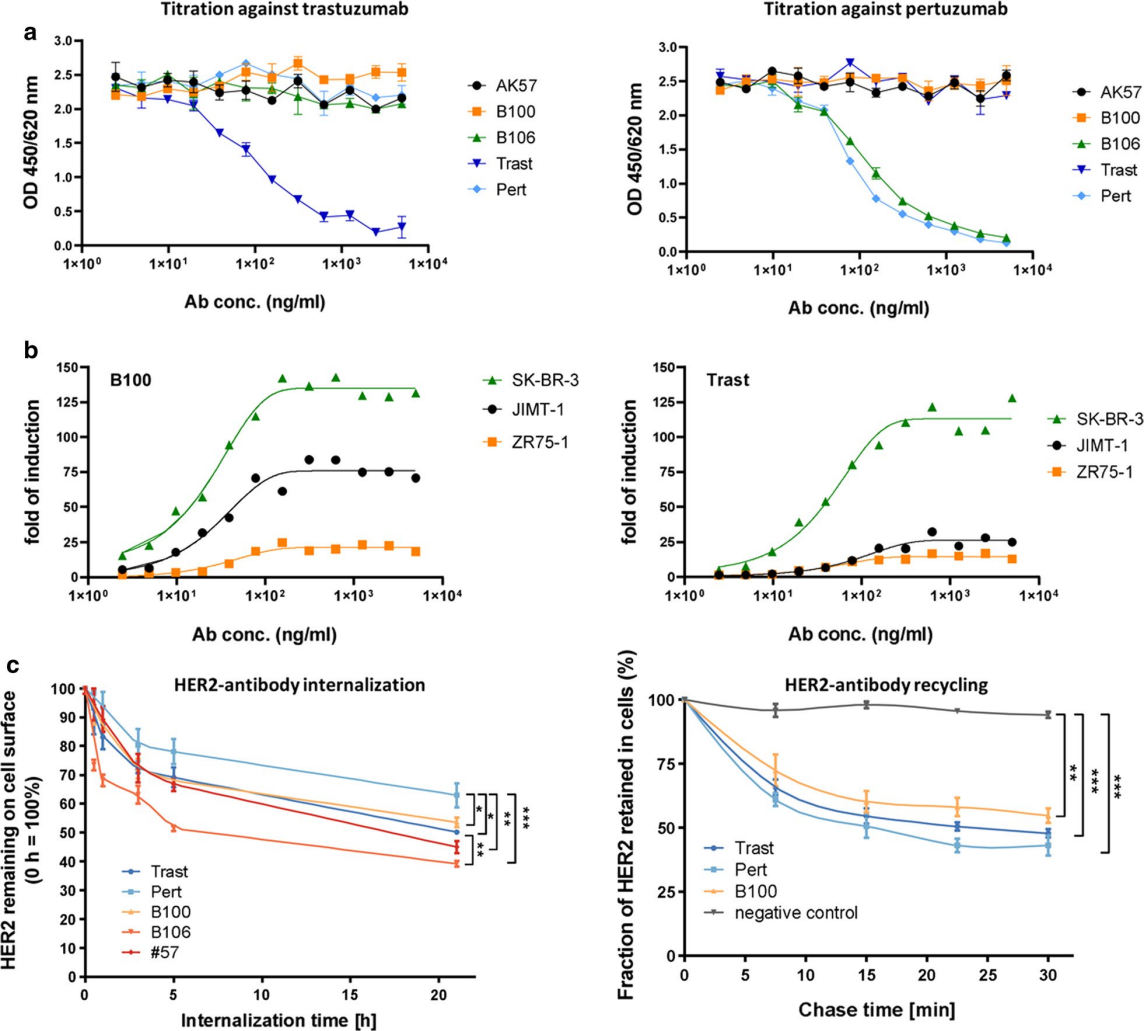
Epitope binding, FcR signaling, internalization and recycling capacity of mAbs. **a** Epitope competition assay of B100, B106, and A57 against trastuzumab (Trast; left graph) or pertuzumab (Pert; right graph) in dependency of antibody concentration is displayed. **b** FcR signaling of B100 (left graph) and trastuzumab (right graph) on JIMT-1 (black), SK-BR-3 (yellow), and ZR-75-1 (green) using different antibody concentrations was determined. **c** Internalization capacity of B100, B106, and A57 in comparison to trastuzumab and pertuzumab on SK-BR-3 cells are displayed: cells were pre-treated with 2 µM Monensin for 2 h to inhibit potential recycling. Amount of HER2 remaining on cell surface were normalized to the amount of HER2 of control cells without treatment, which were set to 100%. Mean ± standard deviation (SD) of three independent experiments are presented. For group wise comparison with Tukey’s multiple comparison post-test was performed (*p ≤ 0.05; **p ≤ 0.01; ***p ≤ 0.001). **d** Recycling capacity of B100 in comparison to pertuzumab and trastuzumab was determined on SK-BR-3 cells. Fluorescence signal at each time point was normalized with the 0 min chase time point signal, which was set to 100%, to calculate the fraction of HER2 retained in cells. Mean ± standard deviation (SD) of three independent experiments are presented. For group wise comparison Tukey’s multiple comparison post-test was performed (*p ≤ 0.05; **p ≤ 0.01; ***p ≤ 0.001)

Epitope mapping in an ELISA binding competition assay revealed no competitive binding of A57, B100, B106, and pertuzumab against trastuzumab (Fig. 2a, left). In addition there was no competition of mAb A57, B100 and trastuzumab binding against pertuzumab binding (Fig. 2a right). Only B106 bound competitively to pertuzumab. In addition to the binding competition assay of AK57, B100, and B106 vs. trastuzumab and pertuzumab we identified the extracellular subdomain III of HER2 as the binding epitope of B100 by another ELISA coated with subdomain specific peptides (data not shown).

The activity in an in vitro Fcγ receptor signaling assay was comparable between trastuzumab and B100 on SK-BR-3 but was higher upon B100 binding to HER2-positive but trastuzumab resistant JIMT-1 cells (Fig. 2b left and right). Trastuzumab and B100 when added to ZR-75-1 cells did not trigger Fcγ signaling which is compatible with the absence or just very low expression of HER2 in this cell type (i.e., no opsonization).

A pronounced internalization of HER2 was found upon A57 (50%), B100 (40%), B106 (55%), and trastuzumab (42%) binding to SK-BR-3 cells. However, pertuzumab treatment did cause only a minor extent of internalization (Fig. 2c left). Recycling capacity after internalization of HER2 on the surface of SK-BR-3 occurred nearly to the same extent of about 40% upon B100, trastuzumab, and pertuzumab treatment (Fig. 2 right). Notably, the whole antibody-receptor-complex was recycled back to the cell membrane while the total amount of HER2 greatly depends on the number of receptors that were internalized before.

### B100 treatment eliminates tumor cells in a trastuzumab resistant Humanized Tumor Mouse model

Mice were generated by transplantation of SK-BR-3 tumor cells with (HTM) or without (TM) the additional transplantation of CD34^+^ HSC derived from CB. HTM were characterized by tumor cell proliferation in the peritoneum (ascites; peritoneal tumor cells = PTC), by their sensitivity or resistance to trastuzumab treatment, and by the amount of metastases formation in different organs [19, 20]. In the age of 9 weeks, treatment with mAb started and was continued for 12 weeks or until animals showed indications of severe sickness. At the end of the experiments spleen and cells isolated from the BM were analyzed for human immune cells repopulation and revealed an average of 52.2% human CD45 in the spleen (mean ±  5.8 SEM; n = 39) and 29.2% human CD45 in the BM (mean ± 5.8 SEM; n = 39; data not shown).

**Fig. 3:**
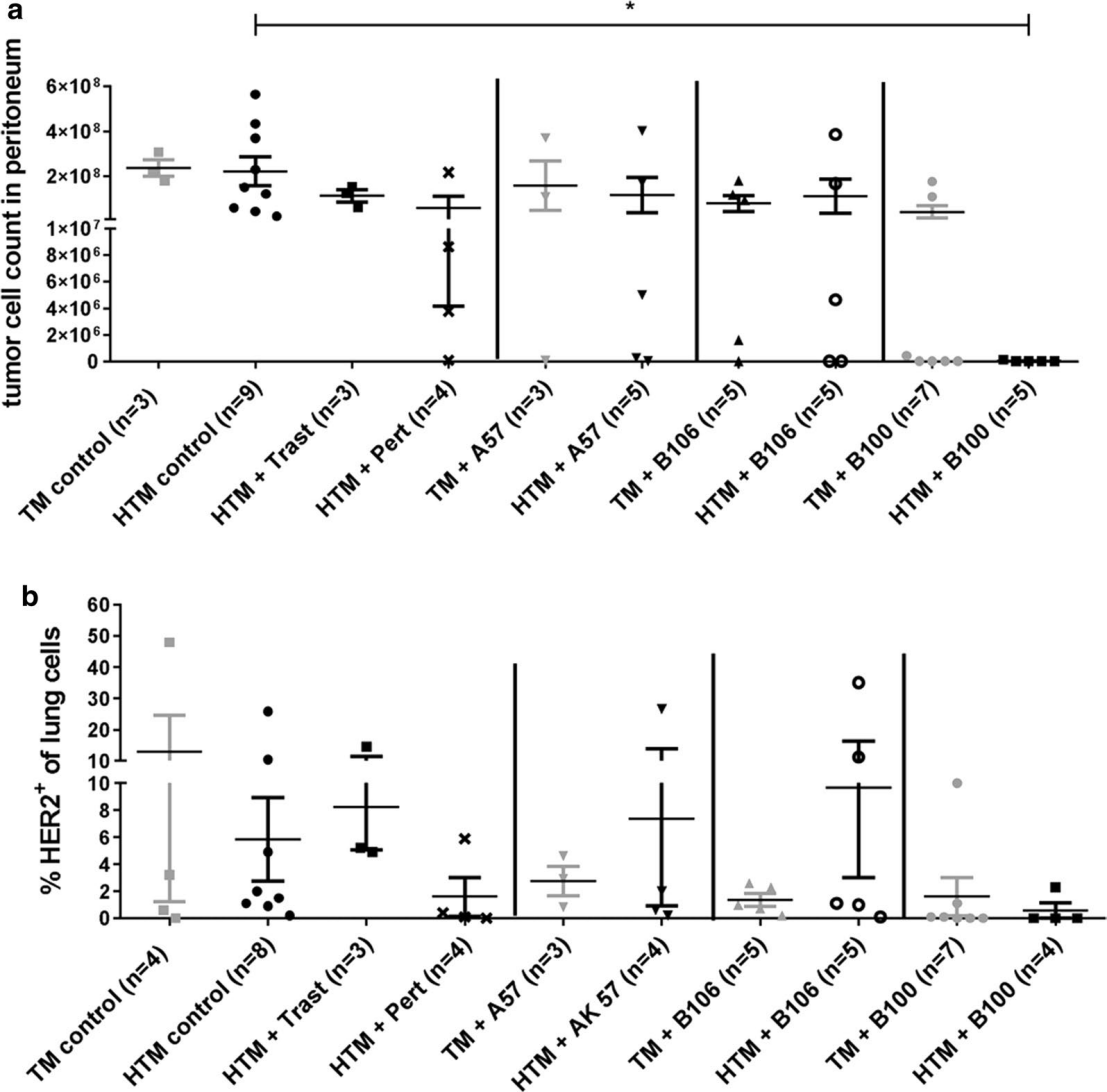
Therapy efficiency of different anti-HER2 antibodies in vivo. **a** Tumor cells isolated from the peritoneum (ascites) of SK-BR-3 transplanted HTM and TM are indicated. **b** Single cells isolated from the lung were stained with anti-HER2 antibody and quantified by flow cytometry. Numbers of animals in each group are indicated in brackets. For statistical analyses Dunnett’s multiple comparisons test was applied and significances are displayed (*p < 0.05). HTM, humanized tumor mice; TM, tumor mice; Trast, trastuzumab; Pert, pertuzumab

At the age of 9 weeks (start of mAb treatment) HTM revealed an amount of about 1.56 × 10^7^ peritoneal tumor cells (mean ± 2.3 × 10^6^ SEM, n = 3, data not shown). The tumor load decreased or increased during the period of mAb treatment. As published previously and reproduced in this study a trastuzumab treatment had no antitumor effect in the SK-BR-3 based HTM model (Fig. 3a). In addition, no significant overall reduction of the tumor burden was detectable in A57 and B106 treated mice. Even though the in vitro effects of A57 and B100 treatments on SK-BR-3 were quite similar (reduction of SPF: A57 > B100; induction of apoptosis: B100 > A57) only the B100 treatment resulted in a total elimination of the tumor cells in the peritoneum of all HTM and in the majority of the TM. Of note, two other mAb were tested in HTM and TM with a high efficiency on SK-BR-3 in vitro (reduction of SPF down to 65% and an induction of apoptosis up to 30%). However, the application of these antibodies in TM and HTM did not result in any treatment efficiency (data not included).

### The treatment of SK-BR-3 based HTM with B100 significantly reduced tumor metastases in the lung, liver, and brain

**Fig. 4:**
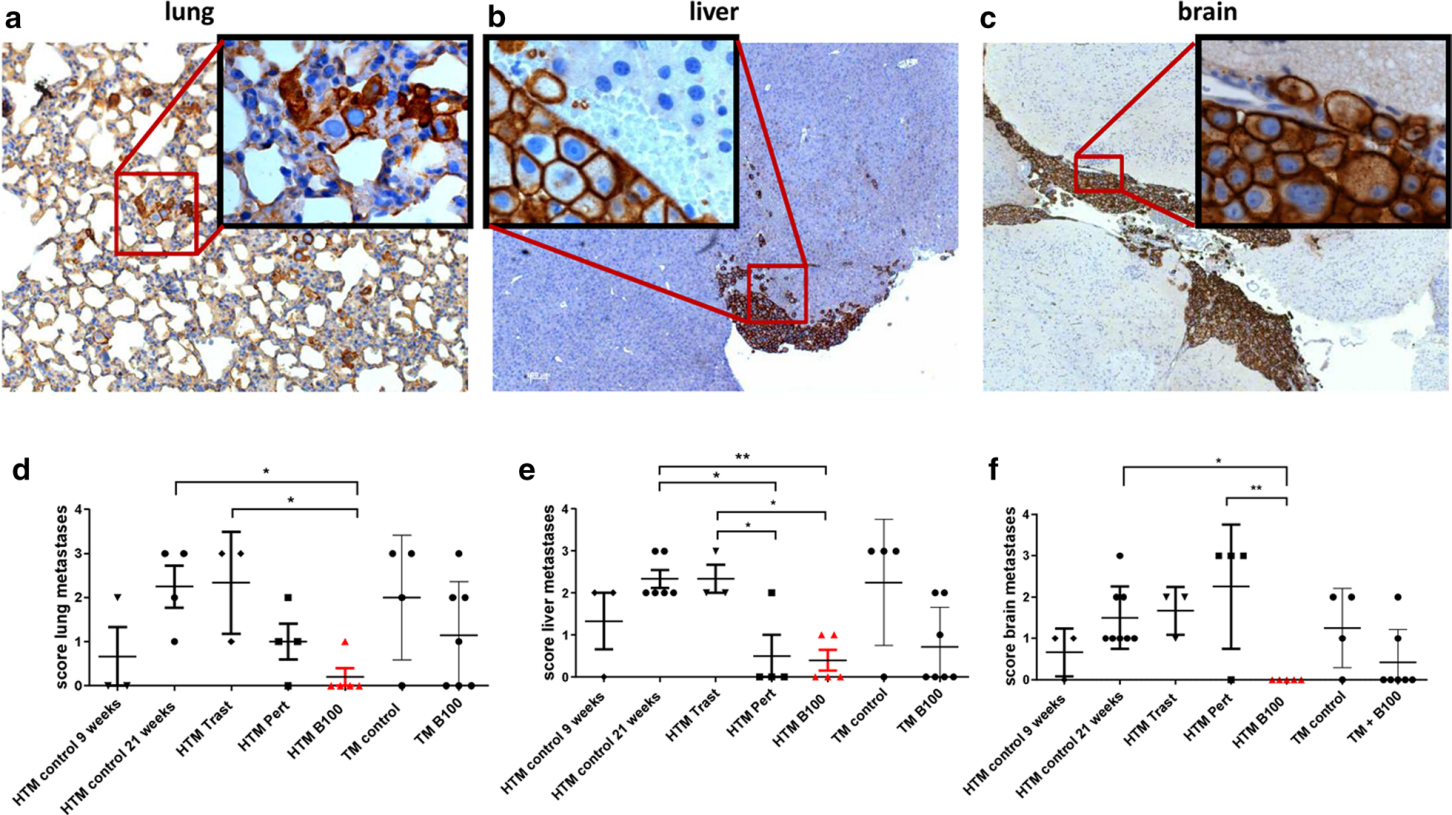
Capacity of anti-HER2 antibodies to inhibit tumor metastases in HTM. Histological sections of lung (**a**), liver (**b**), and brain (**c**) of a HTM are displayed (exemplarily shown for a pertuzumab treated mouse) and metastases scores from the lung (**d**), liver (**e**), and brain (**f**) of untreated HTM (control 9 & ~ 21 weeks), trastuzumab (Trast), pertuzumab (Pert), and B100 treated mice were compared. Each symbol represents one mouse. Data are shown as mean ± SEM and significances were analyzed using Tukey’s multiple comparisons test (*p < 0.05; **p < 0.01). Trast, trastuzumab; Pert, pertuzumab

Flow cytometric analyses of HER2-positive tumor cells in the lung revealed a large variation between the groups treated with individual antibodies but overall no significant reduction in tumor burden in this organ (Fig. 3b). Histological examination of lung, liver, and brain confirmed no significant reduction in tumor load by trastuzumab (Table 2), by A57, or by B106 treatment in HTM or TM (Additional file 2: Table S1). In contrast, the application of pertuzumab resulted in a significantly reduced amount of liver metastases in HTM (Table 2) and the use of B100 significantly reduced metastases in the lung (p = 0.048) and mainly in the brain of HTM (p = 0.0008; Table 2). Moreover, detailed histological examination revealed a significantly decreased metastasis score in the lung (p < 0.05), liver (p < 0.01), and the brain (p < 0.05) in B100 treated HTM compared to untreated HTM (Fig. 4). In particular, the lung and liver tumor scores were significantly reduced in B100 treated HTM compared to the score evaluated for trastuzumab treated mice. A significant reduction of the tumor burden in pertuzumab treated HTM was detectable only in the liver (p < 0.05). However, a significant decline in the tumor score of lung, liver, and brain in B100 treated TM could not be found (Fig. 4).

**Table 2:** Immunohistological assessment of metastases in antibody-treated and non-treated (control) HTM and TM

	9 weeks	21 weeks							
	HTM control	HTM control	HTM Trast	HTM Pert	HTM B100	p-value (control vs. B100)	TM control	TM B100	p-value (control vs. B100)
Lung	1/3	4/4	3/3	3/4	1/5	*0.048*	3/4	4/7	1
Liver	2/3	6/6	3/3	1/4 (*p = 0.0333*)	2/5	0.061	3/4	3/7	0.546
Brain	2/3	8/8	3/3	3/4	0/5	*0.0008*	3/4	2/7	0.242

SK-BR-3 transplanted HTM and TM were immunohistologically stained using anti-HER2 antibodies in the lung, liver, and brain. HTM were analyzed in the age of 9 weeks (start of therapy) and in the age of ~ 21 weeks (end of therapy/experiment). The number of animals with detectable HER2-positive metastasis of the total number of animals (n/n) is indicated. Statistical differences were calculated using the two-sided Fisher’s exact test and significant differences are marked in italics

### B100 treatment of SK-BR-3 based HTM reduced the number of disseminated tumor cells (DTC) in the BM

**Fig. 5:**
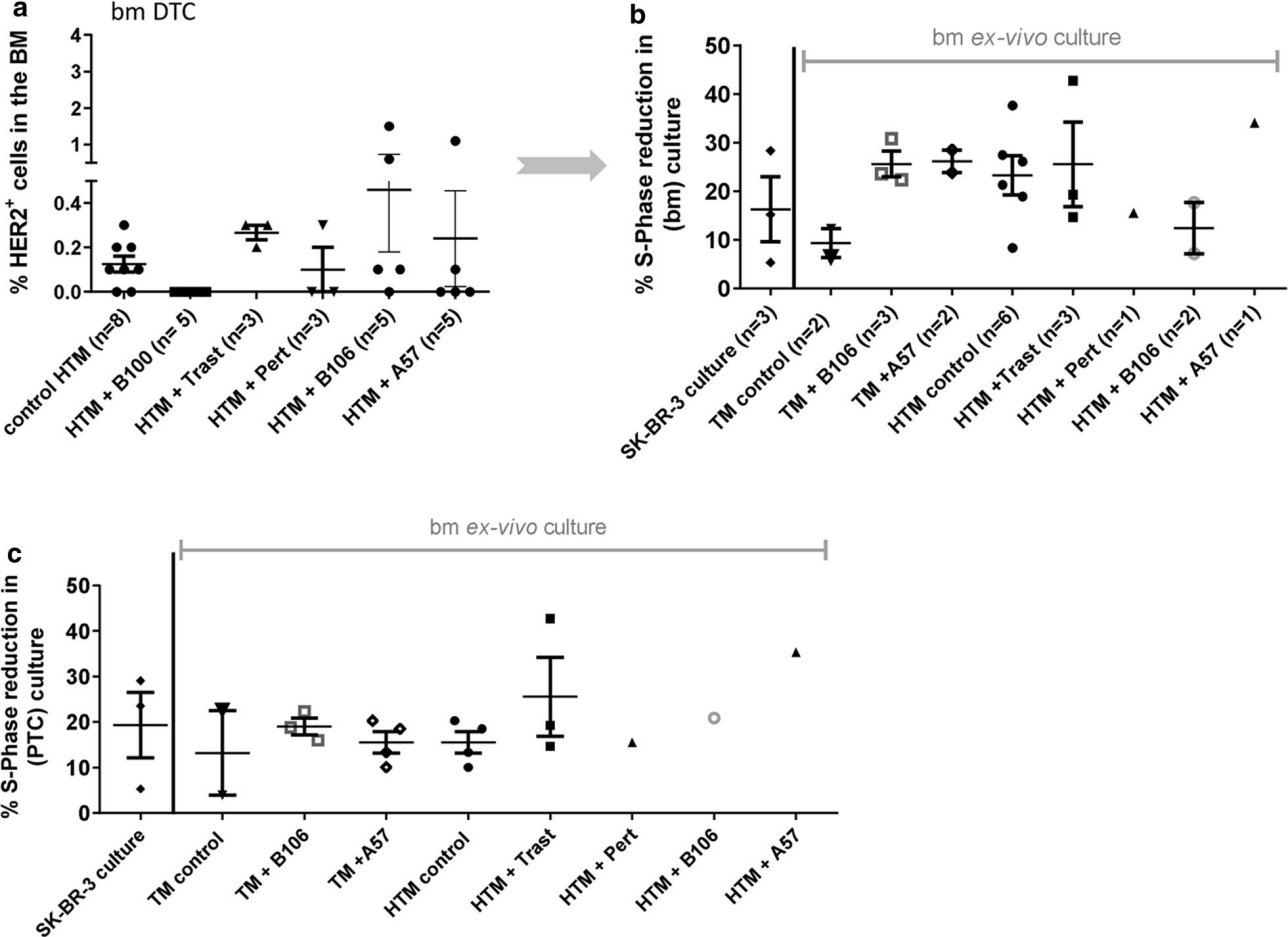
Antibody treatment responsiveness from ex vivo cultured tumor cells isolated from the BM and peritoneum (PTC) of HTM and TM. **a** Percentage of HER2^+^ tumor cells in single cell suspension isolated from the BM of HTM measured by flow cytometry. SPF reduction of trastuzumab treated SK-BR-3 cell cultures in comparison to ex vivo trastuzumab treated tumor cells isolated from BM (**b**) and peritoneum (**c**) of HTM and TM. Each symbol represents one mouse. Data are shown as mean ± SEM and no significances were detectable using Tukey‘s multiple comparison test. Trast, trastuzumab; Pert, pertuzumab

In the age of 9 weeks (start of treatment) only one out of three untreated HTM showed detectable HER2-positive tumor cells (0.1%) in the BM (data not shown). In contrast, single DTCs could be detected in the majority of untreated HTM transplanted with SK-BR-3 breast cancer cells at the end of observation period (Fig. 5a). B100 treatment of HTM prevented the appearance of disseminated HER2-positive tumor cells in the BM. Interestingly, in some of the B106 and A57 treated HTM we found an increased number of DTCs in the BM compared to untreated mice (Fig. 5a). To expand potentially present but undetectable single DTCs extracted from the BM the cell extracts were incubated ex vivo for several weeks (average of 81 days ± 3.95 SEM). Samples, which could be expanded ex vivo were quantified as given in Table 3 and Table S1. At the age of 9 weeks none of the three untreated HTM developed DTC cultures but at the end of the experiment seven out of eight untreated HTM revealed expandable cell cultures. Upon treatment with B100 DTCs from the BM of HTM could not be propagated (0/5), i.e., were not present in the BM (p = 0.0047, Table 3). In contrast a preceding animal treatment with trastuzumab did not impede a subsequent DTC expansion in vitro at all (3/3) whereas from pertuzumab treated animals only one out of four DTC cultures could be propagated (Table 3).

**Table 3:** Success rate of ex vivo propagated DTCs derived from the BM of SK-BR-3-transplanted HTM and TM

	Control	B100	p-value (control vs. B100)	Trast	p-value (control vs. Trast)	Pert	p-value (control vs. Pert)
HTM	7/8	0/5	*0.0047*	3/3	1	1/4	0.067
TM	2/4	0/4	0.4286	nd	–	n. d.	–

Single cells isolated from the femur of SK-BR-3-transplanted HTM and TM were cultured for several weeks to expand DTCs. The number of animals with successfully expanded DTC cultures of the total number of all tested samples (n/n) is indicated. Statistical differences were calculated using the two-sided Fisher’s exact test and significances are typed italic

*nd* not done

### None of the HTM or TM developed trastuzumab resistance in peritoneal or BM derived tumor cells

Successfully expanded DTC cultures were tested for their mAb responsiveness in order to evaluate a potential resistance developed in previously treated animals. Trastuzumab treatment of wild type SK-BR-3 cells typically causes a reduced SPF of about 16.3% (mean ± 6.7 SEM; n = 3, Fig. 5b) compared to untreated controls. However, in the ex vivo expanded DTC cultures from HTM and TM there was a large variation in responsiveness to trastuzumab treatment (Fig. 5b). In B100 treated HTM and TM no disseminated tumor cells could be isolated from the BM. Accordingly, any attempt to expand human tumor cells potentially derived from the BM in these mice in vitro failed (Table 3).

Tumor cells isolated from the peritoneum (PTC) were incubated for 7 days upon ex vivo extraction and subsequently tested for their responsiveness to mAb treatments. When treated with trastuzumab no effect on cell proliferation of the cells could be seen independently from the preceding mAb treatment in vivo (Fig. 5c). B100 treated HTM had no tumor cells in the peritoneum and could therefore not be tested ex vivo (no resistance development possible).

### Resistant trastuzumab treated HTM showed significantly increased B cell fraction and CD4/CD8 T cell ratio in the spleen in B100 treated tumor free HTM

The overall reconstitution with human CD45^+^ cells in the spleen (average of 52.15 ± 5.8 SEM; p > 0.1 in between all groups) and BM (average of 29.21 ± 5.8 SEM; p > 0.1 in between all groups) of untreated, pertuzumab, trastuzumab, and B100 treated HTM were analyzed and revealed no significant differences between treatment groups (data not shown). The level of human reconstitution in the spleen of mAb treated HTM did not depend on the extent of tumor burden (PTC) detected in the peritoneum (p = 0.5045; Pearson’s correlation coefficient r = − 0.1542).

**Fig. 6:**
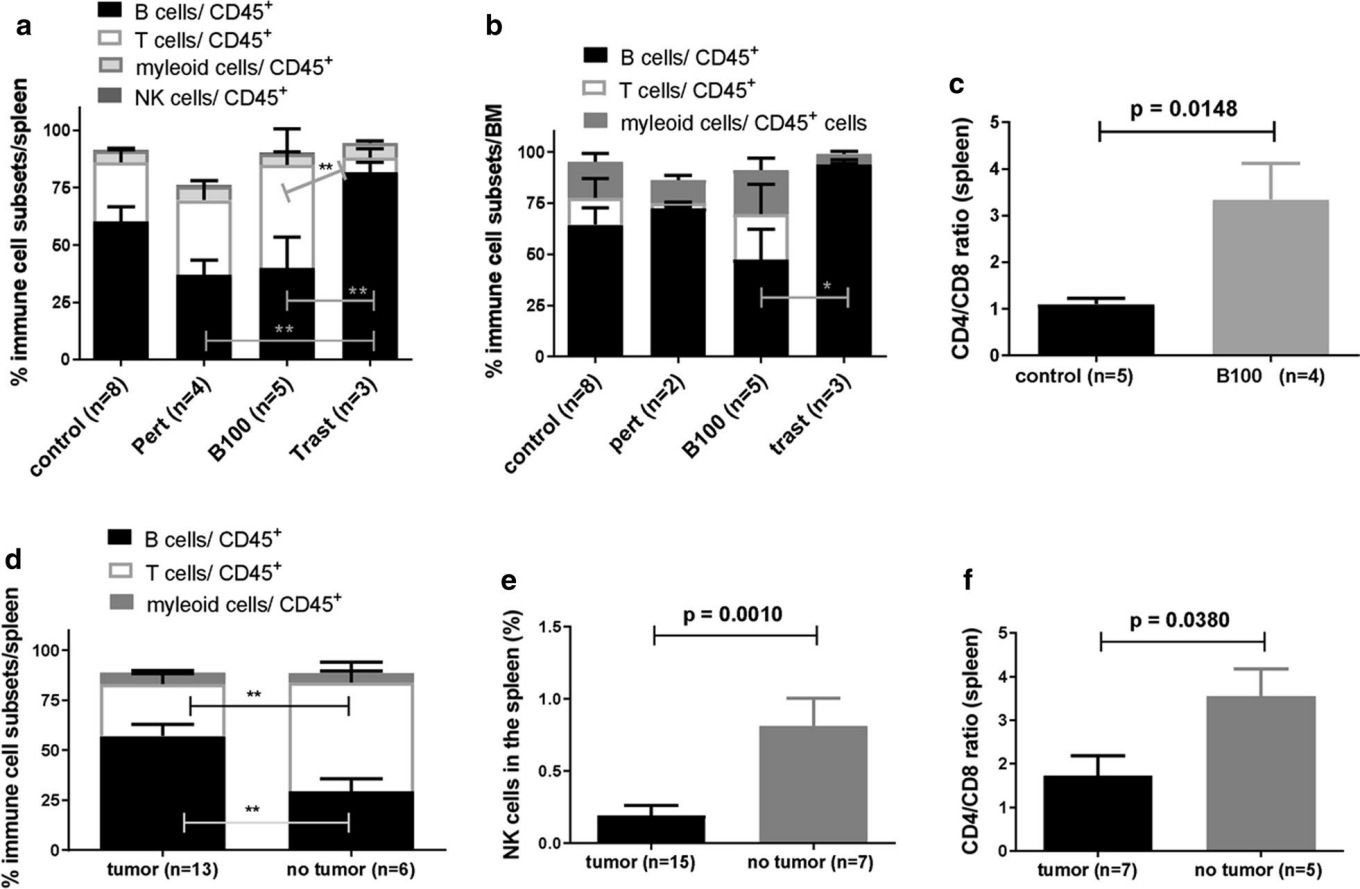
Variations of human immune cell distribution in the spleen and BM of treated and untreated HTM. Immune cell subsets in the spleen (**a**) and BM (**b**) of differently treated HTM are imaged. Data are shown as mean ± SEM and significances were analyzed using Tukey‘s multiple comparison test (*p < 0.05; **p < 0.01). **c** CD4/CD8 ratio of CD3^+^ t cells isolated from the spleen of untreated and B100 treated HTM were compared and significance is indicated (Student’s T-test; p = 0.0148). Percentage of human hematopoietic cells (CD45^+^; **d**), immune cell subsets (**e**) and CD4/CD8 ratio (**f**) of HTM in the spleen of responder (no tumor) and non-responder (tumor) of all mAb treated mice. Significant difference was calculated by Student’s T-test (p = 0.0197). Trast, trastuzumab; Pert, pertuzumab

However, human immune cell populations in HTM are composed by a significant higher B cell fraction in the spleen of trastuzumab (unresponsiveness) treated HTM versus pertuzumab (p < 0.05) and B100 (p < 0.05) treated HTM (Fig. 6a). In addition, a significant higher fraction of B cells was also found in the BM of trastuzumab treated cells vs. B100 treated mice (Fig. 6b). However, there was no significant difference of myeloid or NK cell quantity detectable between the groups. Remarkably, B100 treated HTM showed a significantly increased CD4/CD8 ratio compared to the control (Fig. 6c; p = 0.0148). The amount of CD45-positive human immune cells infiltrating into the peritoneum was slightly increased in B100 treated HTM (mean 10.35 ± 4.7 SEM) compared to pertuzumab treated (mean 5.13 ± 4.1 SEM) and to control HTM (mean 2.35 ± 1.5 SEM; Figure S2A). Interestingly, trastuzumab treated HTM did not show any immune cells in the peritoneum. However, independent of the antibody treatment the majority of detected immune cells belonged to the T cell fraction (Figure S2B).

Next we evaluated if the general reconstitution of human immune cells or the proportion of T cells (in animals independently with which antibody treated) might be an indicator for responsiveness. Indeed HTM without tumor at the end of mAb treatment showed a significant higher T cell (and lower B cell) proportion in the spleen (Fig. 6d). Moreover, HTM without tumors showed significant higher amounts of NK cells (Fig. 6e) in the spleen and an increased CD4/CD8 ratio (Fig. 6f).

## Discussion

Here we investigated the treatment efficiency of HER2-positive but trastuzumab insensitive BC cells both in vitro and in vivo using HTM. While an enhanced HER2 expression in SK-BR-3 cells can be attributed to a pronounced gain of the her2 gene (her2 gene copy number on average > 10 per cell, gene/centromere ratio 4.8), the frequency of her2 gene copies in MDA-MB-361 and MD-MB-453 cells is only moderately elevated (about 5 gene copies per cell; her2/centromere ratio 3.6 and 2.1, respectively) [25]. Thus, all three BC cell lines are by diagnostic definition HER2-positive, however differ in terms of the her2 gene dose. Therefore, the resistance of MDA-MB-361 and especially MD-MB-453 cells to trastuzumab treatment is most likely due to the insufficient HER2 receptor content since trastuzumab has been repeatedly shown to impair the growth of tumor cells only if the HER2 expression is considerably elevated [26–28]. Nevertheless, MDA-MB-361 cells do slightly respond to the treatment with the B100 and B106 mAbs. More importantly, SK-BR-3 cells that do almost not respond to a treatment with trastuzumab and pertuzumab appear sensitive to all other treatments with rabbit derived antibodies used as stand-alone applications, i.e., B100, B106, A57. This finding is also valid for all dual antibody targetings tested. Thus, compared to trastuzumab a number of other rabbit derived antibodies were used and showed enhanced treatment efficiency in SK-BR-3 cells in vitro. Thereby, the emergence of a significant cohort of apoptotic tumor cells (> 30% in SK-BR-3) which is not seen upon trastuzumab treatment is the most striking effect observed upon BC cell exposition to B100 in vitro. In the entire view, an enhanced B100 treatment efficiency in vitro can be attributed to an attenuated cell proliferation but predominantly to the induction of apoptotic cell death.

As repeatedly demonstrated, the treatment efficiency of HER2-positive BC cells using different anti-HER2 antibodies varies considerably. This phenomenon can be attributed to a variety of mechanisms, amongst them to a diverse molecular profile of predictive biomarkers in tumor cells but also to different binding epitopes and binding affinities. As shown for trastuzumab that binds to the extracellular HER2 domain IV pertuzumab recognizes domain II that is directly involved in ligand dependent and independent interaction of HER2 at the cell surface. Thus, a specific epitope recognition can directly affect the receptor activity but can also facilitate or impair receptor dimerization and subsequent cross-activation due to steric hindrances. Importantly, the binding studies with B100, trastuzumab, and pertuzumab revealed a non-competitive binding of these three immunoglobulins, which allows the combined use of two (or even three) of these antibodies for therapy purposes in the clinical setting. Advanced epitope mapping of B100 proved a docking to receptor domain III with a binding efficiency that is—when bound to SK-BR-3 cells—higher than that of trastuzumab and pertuzumab. This feature is possibly due to the different epitope binding and is most likely one parameter that contributes to the high treatment efficiency in vitro [29]. A synergistic treatment effect of two anti-HER2 antibodies binding to different epitopes has not only been demonstrated for trastuzumab and pertuzumab in BC but also for other antibody combinations used for the treatment of additional malignancies [30]. In contrast, the HER2 turnover (i.e., receptor internalization and recycling) upon trastuzumab and B100 binding differs just slightly and is probably not part of individual treatment efficiencies caused by these immunoglobulins. A comparatively low degree of HER2 internalization is only seen upon pertuzumab binding whereas the use of pertuzumab alone is less efficient than the administration of trastuzumab, both in the preclinical and clinical setting.

Apart from the in vitro studies the most striking treatment efficiency upon B100 administration could be observed in HTM. Compared to trastuzumab and pertuzumab treatments, the application of B100 to HTM prevented the formation of lung and liver metastasis nearly completely. Conspicuously absent is the formation of brain metastases in any of the B100 treated HTM that totally distinguishes this group from all HTM groups treated with other antibodies and from TM treated with B100. This finding is in perfect accordance with the efficient prevention of tumor cell dissemination or the complete eradication of tumor cells in the BM of HTM, respectively. Thus, tumor progression by seeding and outgrowth of tumor cells at distant sites is efficiently blocked in HTM by B100 treatment. Overall, the treatment studies based on HTM convincingly demonstrate the enhanced anti-tumor activity of B100 in vivo. In addition, the use of HTM allows to exclude antibodies which show remarkable treatment efficiency in vitro but insufficient activity under human like condition (e.g., A57) from further evaluation and in particular from prospective clinical trials in humans. Worth to mention is that due to their human like characteristics HTM are becoming increasingly important in the field of preclinical treatment studies. The value of mice with a human immune system to evaluate treatment success, failure and side effects involved has been recently highlighted by the US Food and Drug Administration [31, 32].

Importantly, in TM (i.e., in the absence of human immune cells) the B100 treatment efficiency was not nearly as high as in HTM (i.e., in the presence of human immune cells). Not only the growth of SK-BR-3 BC cells in the peritoneum is completely prevented but the formation of metastasis in different organs (e.g., lung, liver) is also significantly reduced or even totally absent (brain). This finding can be taken as a strong evidence that in vivo B100 does not only have a cellular anti-tumor effect but also has the capacity to stimulate an immunological tumor defense, for example via Fc mediated ADCC. Additional evidence for an immunological tumor defense is given by an increased T cell fraction in the spleen of B100 treated mice for example compared to the immune cell distribution seen in trastuzumab treated HTM. More specifically, the CD4/CD8 T-cell ratio was significantly increased by the administration of B100 to HTM. We previously described a preferred CD4 T cell activation over an activation of cytotoxic T cells in HTM both without [19] and with antibody (trastuzumab) treatment [20]. Thus, a cytotoxic CD4 cell activity triggered by the application of therapeutic antibodies seems to play a relevant role in HTM. An important role of CD4 T cells in cancer defense has been formerly outlined elsewhere [33]. Mechanistically, CD4 T cells may directly kill the tumor cells by recognition of MHC II presented antigens which causes the release of lytic enzymes as perforin and granzyme [34] or FAS/FAS ligand induced cell death [35]. In addition, CD4 T cells can indirectly kill tumor cells via directly activating antigen presenting cells (APC). Activated APCs have in turn the capacity to increase the cross-priming of tumor-specific cytotoxic T lymphocytes (CTL) or can eliminate tumor cells for example by the release of nitric oxide [36].

Furthermore is to mention that in HTM without detectable tumor cells after antibody treatment the NK cell fraction was significantly increased. We experimentally demonstrated here a significant capacity of the rabbit derived and chimerized B100 to Fc-receptor signaling in vitro, a mechanism that seems to work in HTM as well, even though there was a pro-apoptotic in vitro activity even in the absence of any immune cells. Taken together, CD4 T and NK cells seem to play a relevant role for a successful anti-tumor antibody therapy in SK-BR-3 based HTM.

With the help of HTM we monitored an extraordinary treatment efficiency under human like conditions when B100 was applied for the targeting of HER2-positive BC cells which are poorly responsive to trastuzumab treatment. The most relevant finding is the prevention of tumor cell dissemination and formation of metastases. Demonstrably, the binding of B100 induces apoptotic cell death in vitro an effect that might contribute to the tumor cell elimination in vivo as well. Thus the anti-proliferative effect might curb solid tumor growth whereas the elimination of DTCs and tumor cells at distant sites might be attributed to a pro-apoptotic activity of B100 potentially mediated both by direct cellular effects and by the recruitment of immune (e.g., NK/CD4) cells. The eradication of those cells might in turn prevent the generation and selection of dormant cells with a stem cell phenotype that emerge in SK-BR-3 based HTM without B100 treatment as previously shown [20]. In particular, those dormant cells would most likely become resistant to cytotoxic treatments [37]. Overall, the dual mechanism of action might be the basis for the extraordinary treatment efficiency seen in HTM.

In the meantime, we turned the chimeric B100 mAb into a humanized version. First analyses indicate that the humanized mAb does not come with any loss of treatment efficiency. Extended analyses based on the humanized mAb B100 version using HTM (and potentially other trastuzumab resistant preclinical models) are required before the immunoglobulin can be transferred to clinical trials in order to verify the treatment efficiency in humans as well. Different treatment scenarios appear reasonable: Due to its dual therapeutic activity in HTM a humanized B100 version might show therapeutic efficiency in patients with advanced (i.e., metastasized) BC as well. However, humanized B100 might also be useful for combination treatments (with trastuzumab) of early BC in order to improve the treatment response rate in the neoadjuvant setting. For example, while trastuzumab drives tumor cells into quiescence and thereby reduces the sensitivity to anti-proliferative cytotoxic treatments [38–40] B100 could complement this anti-proliferative effect by driving (quiescent) cells into apoptotic death. Therefore, a neoadjuvant cytotoxic treatment with adequately and sufficiently dosed and humanized B100 in combination with trastuzumab promises to develop synergistic treatment effects. Overall, it can be expected that well established anti-HER2 treatments both for early and advanced BC can be improved by the application of novel strategies and the integration of new reagents with unique mechanistic features. The rabbit derived B100 immunoglobulin has the potential to become part of an extended portfolio compiled for the fight against HER2-positive malignancies, in particular those that show insufficient response to established treatment regimens.

## Conclusion

Overall, we present here a novel chimeric monoclonal highly efficient anti-HER2 antibody named “B100” that comes with pronounced anti-tumor activity both in vitro and in a preclinical HTM in vivo model. The most striking feature of B100 is its pro-apoptotic activity and its capacity to almost completely eradicate human HER2-positive BC cells in HTM including brain metastases. The validation of the treatment efficiency of a “humanized B100” in HTM and other relevant preclinical models and finally the translation to the clinical setting is warranted and especially promising for the treatment of trastuzumab resistant BC.

## Supplementary information

**Additional file 1: Figure S1.** Fitting curves for domain specific ELISAs. A: B100 (R squared = 0.8412), B: trastuzumab (R squared = 0.7954), C: pertuzumab (R squared = 0.8286).

**Additional file 2: Table S1.** Immunohistological assessment of metastases in antibody-treated and non-treated (control) HTM and TM. Lung, liver, and brain of SK-BR-3 transplanted HTM and TM were immunohistologically stained using an anti-HER2 antibody. HTM were analyzed in the age of ~ 21 weeks (end of therapy/experiment). The number of animals with detectable HER2-postive metastasis of the total number of animals (n/n) is indicated.

**Additional file 3: Figure S2.** Immune cell infiltration into the peritoneum of treated and untreated HTM. The percentage of CD45-positive human hematopoietic cells (A) and the immune cell subsets (B) infiltrated into the peritoneum of HTM are presented. The numbers of animals in each group are indicated in brackets. Trast = trastuzumab; Pert = pertuzumab.

## Data Availability

All data generated or analyzed during this study are included in this published article [and its additional files].

## Abbreviations

ADCC: antibody-dependent cell-mediated cytotoxicity
APC: antigen presenting cells
BC: breast cancer
BM: bone marrow
CB: cord blood
CTL: cytotoxic T lymphocytes
GALT: Gut-Associated Lymphoid Tissues
HER2: human epidermal growth factor receptor 2
HSC: hematopoietic stem cells
HTM: humanized tumor mice
mAbs: monoclonal antibodies
NK: natural killer
TM: tumor mice
VDJ: variable, diversity, and joining
