# Supplementary figures and images for: A novel rabbit derived anti-HER2 antibody with pronounced therapeutic effectiveness on HER2-positive breast cancer cells in vitro and in humanized tumor mice (HTM)
Source: J Transl Med. 2020 Aug 15;18:316. doi: 10.1186/s12967-020-02484-9 (PMC7429704; doi:10.1186/s12967-020-02484-9)

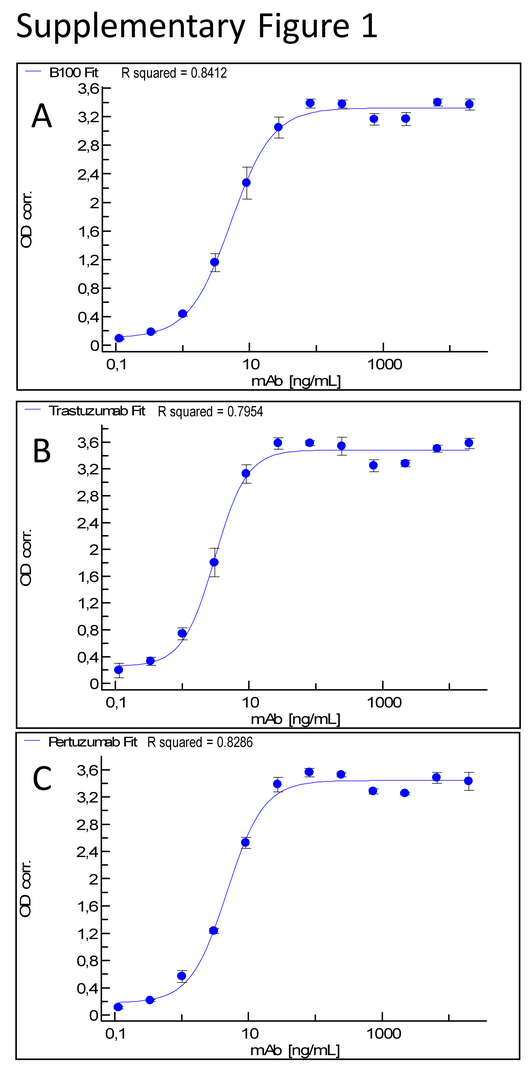

Supplement: Supplementary file 1 — Additional file 1: Figure S1. Fitting curves for domain specific ELISAs. A: B100 (R squared = 0.8412), B: trastuzumab (R squared = 0.7954), C: pertuzumab (R squared = 0.8286). [file 12967_2020_2484_MOESM1_ESM.tif]

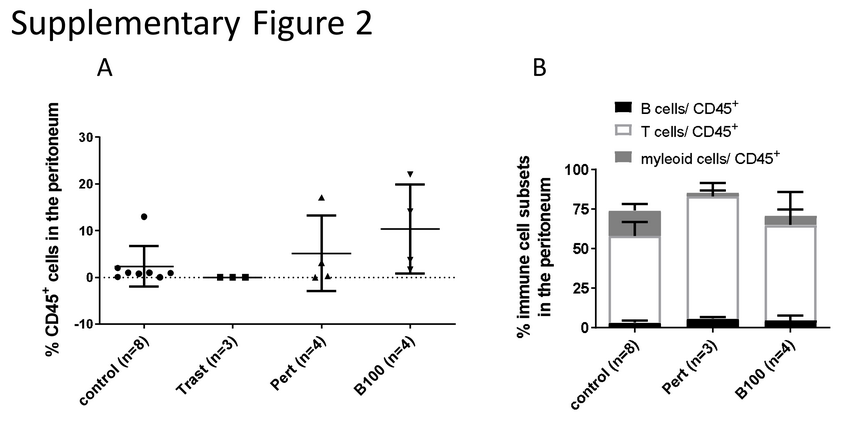

Supplement: Supplementary file 3 — Additional file 3: Figure S2. Immune cell infiltration into the peritoneum of treated and untreated HTM. The percentage of CD45-positive human hematopoietic cells (A) and the immune cell subsets (B) infiltrated into the peritoneum of HTM are presented. The numbers of animals in each group are indicated in brackets. Trast = trastuzumab; Pert = pertuzumab. [file 12967_2020_2484_MOESM3_ESM.tif]
