# Supplementary material for: A novel rabbit derived anti-HER2 antibody with pronounced therapeutic effectiveness on HER2-positive breast cancer cells in vitro and in humanized tumor mice (HTM)
Source: J Transl Med. 2020 Aug 15;18:316. doi: 10.1186/s12967-020-02484-9 (PMC7429704; doi:10.1186/s12967-020-02484-9)
Supplement: Supplementary file 2 — Additional file 2: Table S1. Immunohistological assessment of metastases in antibody-treated and non-treated (control) HTM and TM. Lung, liver, and brain of SK-BR-3 transplanted HTM and TM were immunohistologically stained using an anti-HER2 antibody. HTM were analyzed in the age of ~ 21 weeks (end of therapy/experiment). The number of animals with detectable HER2-postive metastasis of the total number of animals (n/n) is indicated. [file 12967_2020_2484_MOESM2_ESM.docx]

Table S1: Immunohistological assessment of metastases in antibody-treated and non-treated (control) HTM and TM. Lung, liver, and brain of SK-BR-3 transplanted HTM and TM were immunohistologically stained using an anti-HER2 antibody. HTM were analyzed in the age of ~ 21 weeks (end of therapy / experiment). The number of animals with detectable HER2-postive metastasis of the total number of animals (n/n) is indicated.

| **HTM** | | | | | **TM** | | |
| --- | --- | --- | --- | --- | --- | --- | --- |
|  | **HTM control** | **Trast** | **A57** | **B106** | **TM control** | **A57** | **B106** |
| **lung** | 4/4 | 3/3 | 3/3 | 2/4 | 3/4 | 2/2 | 2/3 |
| **liver** | 6/6 | 3/3 | 2/3 | 2/3 | 3/4 | 3/3 | 3/4 |
| **brain** | 8/8 | 3/3 | 1/3 | 3/5 | 3/4 | 2/3 | 3/4 |
| **DTC culture** | 7/8 | 3/3 | 1/4 | 2/5 | 2/4 | 2/3 | 3/5 |
